# MicroGen: a MIAME compliant web system for microarray experiment information and workflow management

**DOI:** 10.1186/1471-2105-6-S4-S6

**Published:** 2005-12-01

**Authors:** Sarah Burgarella, Dario Cattaneo, Francesco Pinciroli, Marco Masseroli

**Affiliations:** 1 BioMedical Informatics Laboratory, Bioengineering Department, Politecnico di Milano, piazza Leonardo da Vinci 32, 20133 Milan, Italy

## Abstract

**Background:**

Improvements of bio-nano-technologies and biomolecular techniques have led to increasing production of high-throughput experimental data. Spotted cDNA microarray is one of the most diffuse technologies, used in single research laboratories and in biotechnology service facilities. Although they are routinely performed, spotted microarray experiments are complex procedures entailing several experimental steps and actors with different technical skills and roles. During an experiment, involved actors, who can also be located in a distance, need to access and share specific experiment information according to their roles. Furthermore, complete information describing all experimental steps must be orderly collected to allow subsequent correct interpretation of experimental results.

**Results:**

We developed *MicroGen*, a web system for managing information and workflow in the production pipeline of spotted microarray experiments. It is constituted of a core multi-database system able to store all data completely characterizing different spotted microarray experiments according to the Minimum Information About Microarray Experiments (MIAME) standard, and of an intuitive and user-friendly web interface able to support the collaborative work required among multidisciplinary actors and roles involved in spotted microarray experiment production. *MicroGen *supports six types of user roles: the researcher who designs and requests the experiment, the spotting operator, the hybridisation operator, the image processing operator, the system administrator, and the generic public user who can access the unrestricted part of the system to get information about *MicroGen *services.

**Conclusion:**

*MicroGen *represents a MIAME compliant information system that enables managing workflow and supporting collaborative work in spotted microarray experiment production.

## Background

Microarray systems presently represent the most diffuse high-throughput technology in the biomolecular field. Among them, spotted cDNA microarrays are widely diffused both in single research groups and in biotechnology service centres because of their flexibility and lower running costs. However, they inherently require a few different technical skills and involve several articulated experimental steps, with numerous critical experimental parameters that must be carefully complied in order to ensure reliable and comparable results. Thus, complete information describing all experimental steps must be orderly collected to allow correct subsequent interpretation of experimental results. For these reasons, spotted microarray experiments tend to be produced in central facilities rather than in single laboratories. Furthermore, different actors, who can also be located in a distance, often take part in a microarray experiment, ensuring all required skills. In such cases, they need to access and share specific experimental information according to their skills and role in the experiment, thus they act in a typical collaborative work scenario.

To standardize the considerable amount of heterogeneous information and data produced in a microarray experiment in order to allow their portability and comparability, the Microarray Gene Expression Data (MGED) society proposed a standard called Minimum Information Amount about Microarray Experiments (MIAME) [1,2]. It precisely defines the information that must be collected during microarray experiment production to completely define experimental and array design, experimental procedures, and generated data results. MIAME standard has greatly standardized presentation of microarray results and allowed aggregation and comparison of results from different centres within common public repositories such as ArrayExpress, GEO, and SMD [3-5].

To facilitate management and local storage, according to the MIAME standard, of great quantity of microarray data produced in single laboratories or research centres, a few software products are available [6-11]. However, they mainly focus on maintaining data integrity [6] in a flexible and robust database environment [7] directly compatible with production instrumentation platforms and in facilitating data analysis [8]. Very few of them limitedly consider a possible ideal production workflow [9], whereas at present to our knowledge none of them support collaborative work in microarray experiment production. Specifically focusing on these last two aspects, we developed *MicroGen*, a MIAME compliant web-based information system for managing all the information completely characterizing spotted microarray experiments and the produced data. Based on experiment workflow, it supports distributed collaborative work in the production pipeline of spotted microarray experiments.

## Results

### Database

*MicroGen *is a MIAME compliant multi-database information system for the workflow management of the production pipeline of spotted microarray experiments.

Its core relational database is designed according to the MIAME standard and gathers information of each performed experiment. In it, according to the experimental workflow, different sets of database tables have been implemented to store descriptions of experiment design, used samples, preparation extraction and libelling, array design, hybridisation procedures and parameters, measurement information and specifications. In accordance with the MIAME specifications, the following sets of information are structured within the database tables. The *experiment design *set includes: type of experiment (e.g. comparison of normal versus pathologic tissue), experimental factors (parameters or conditions tested), hybridisation design, number of hybridisations performed in the experiment, type of reference used for the hybridisations, performed quality control steps, and URL of any websites containing additional experiment information. The *used biological samples, extraction preparation, and labelling *set includes: origin of the biological sample (organism and sample provider name), its characteristics (gender, age, developmental stage and disease state), manipulation of biological samples and used protocols (growth conditions, treatments and separation techniques), protocols for preparing the hybridisation extract (RNA or DNA extraction and purification), labelling protocol, and used external controls (spikes). The *array design *set includes: platform type, surface and coating specifications, PCR amplification, commercial availability of the arrays, protocols of spotting, and information of additional treatments performed. The *hybridisation procedures and parameters *set includes: protocols and conditions used during hybridisation, blocking and washing. Finally, the *measurement data and specifications *set includes: type of used scanning hardware and software, used image analysis software and type of performed image quantifications, and description of measurements produced by the image-analysis software. A comprehensive view of all considered MIAME entries is reported in Table 1.

Other database tables have been implemented in order to collect data regarding all people, biologists or technicians, who take part in each experiment.

All data regarding clones available for spotting (i.e. type, name, identification code, and characteristics) are orderly stored in additional databases customisable according to the types of used microarrays (e.g. medium or high density microarrays).

### Actors and experimental workflow

In *MicroGen *we modelled six types of users, each with his/her own functionalities and privileges. The first four types correspond to the different actors possibly involved in the production of spotted microarray experiments, and the functionalities made them available were modelled according to the experimental workflow. These actors include:

- the *researcher *who wants to perform the microarray experiment (a biologist or a medical doctor who asks specialized biotechnology technicians for microarray production and hybridisation);

- the *spotting operator *(a technician specialized in microarray production and spotting);

- the *hybridisation operator *(a technician specialized in microarray hybridisation);

- the *image processing operator *(a technician specialized in analysis of generated microarray images and in production of quantification results).

The last two modelled *MicroGen *users are:

- the generic *public user, *who can read system service presentation and a tutorial of its use, and can download an example pdf document (see Additional file 1), automatically compiled by the system according to the MIAME standard, which describes a performed sample microarray experiment);

- the *web master *who manages users and registrations to the system, and has access to statistical data regarding performed experiments managed by the system.

In order to access *MicroGen *system functionalities, the first four kinds of users must register to the system. A registered *researcher *has then three possibilities: 1) define a new experiment, 2) verify the progress of requested experiments, and 3) consult the fully compiled MIAME description and the quantitative data of concluded experiments (for an example, see Additional file 1).

When the first option is selected, the researcher must define the general specifications about the experiment he/she wants to perform by entering the information required according to the MIAME standard (Table 1). Then, the clone libraries available for spotting the required microarrays can be selected. All the information about the clones chosen to be spotted on the microarrays is saved in a labelling excel file automatically generated by the system and easily downloadable by the researcher. Finally, the researcher must describe the required hybridisation design, in particular origin and manipulation of used biological samples.

Also a registered *spotting operator *has three, but different, possibilities to choose from: 1) receive the request of a new experiment and start the creation of new microarrays according to their descriptions in the microarray labelling file previously generated by the researcher; 2) after spotting the required microarrays, complete his/her task by entering in the system all information about the performed experimental step according to the MIAME standard (Table 1); 3) consult the fully compiled MIAME description of concluded experiments (for an example, see Additional file 1).

A registered *hybridisation operator *has three options as well: 1) receive new spotted microarrays and start their hybridisation, in case consulting the related experiment MIAME information compiled till that experimental step and the microarray labelling file; 2) after hybridising the spotted microarrays and scanning them in order to acquire their images, complete the hybridisation procedure by entering in the system all information about the performed experimental step according to the MIAME standard (Table 1).

3) upload on *MicroGen *web server the produced microarray images. This last option also allows consulting the fully compiled MIAME description of the concluded experiments (for an example, see Additional file 1).

Also a registered *processing operator *has three different options: 1) receive the acquired images of a new experiment that need to be processed and quantified; 2) after downloading, processing and quantifying the acquired microarray images, and producing their quantification files, upload these last in a central system repository and complete the quantification step by entering, according to the MIAME standard, the specifications about instrumentation and software used for the quantifications; 3) consult the fully compiled MIAME description of the concluded experiments (for an example, see Additional file 1). When a *processing operator *completes all steps in his/her option 2), the whole experiment is completed and the system automatically sends an informative e-mail to the researcher who requested the experiment.

In order to manage the workflow of each specific experiment and make automatically available to the involved actors the information they need at the right time when they require it, *MicroGen *assigns a status code to each managed experiment. Value of the current status and date of each previous status change are saved in each experiment workflow log together with identifiers of all actors that took part in the experiment. Seven different experimental statuses have been defined: 1 = experiment required by a *researcher *but not started yet, 2 = experiment started by a *spotting operator*, 3 = microarray spotting completed, 4 = experiment taken by a *hybridisation operator*, 5 = hybridisation completed, 6 = experiment taken by a *processing operator*, 7 = experiment completed.

## Discussion

Three are the main issues related to information management and workflow support in the production of microarray experiments: the large amount of information produced, their heterogeneity, and the geographic distance that may exist among different actors working on a same experiment. Thus, a system designed to perform the tasks related to information management and workflow support, must grant flexibility, consistency and completeness in managing experimental workflow and correct storage of all produced information. Moreover, to support at the same time collaborative activities, it must be easily accessible to users geographically distant and provide them the information they need when they require it. Such information management can be achieved with appropriate architecture's design of the system database, which also gives better performances of the whole database. For example, if some data that are likely to be consulted at the same time are stored in the same table, less joins among database tables are necessary to extract the required data, thus database interrogation time is reduced. Furthermore, if all information about a performed microarray experiment is completely and orderly collected, it can be used to possibly improve analysis results of produced experimental data and to compare data from different performed experiments.

**Figure 1:**
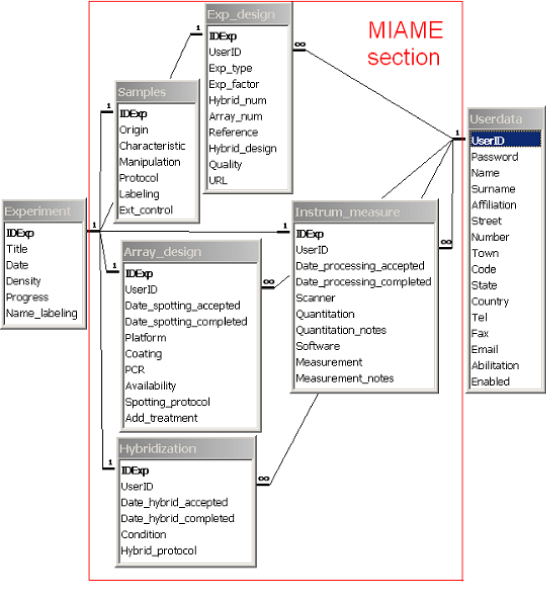
MIAME section tables of *MicroGen *core relational database.

Taking into account the above issues, the architecture of *MicroGen *core database has been carefully designed and experimental information has been structured in tables closely reflecting MIAME standard sections (Figure 1).

**Figure 2:**
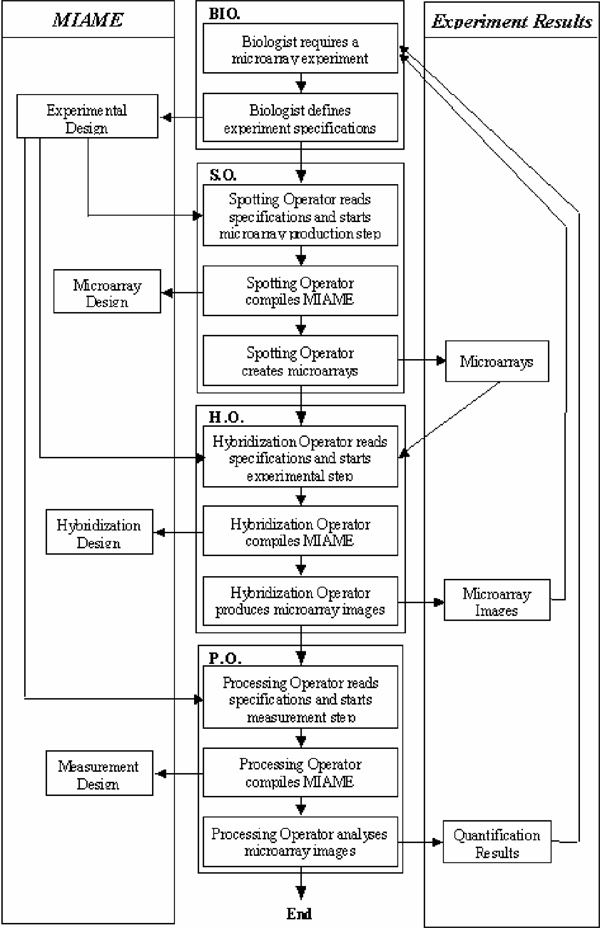
**Experimental workflow, shared information and actors of microarray experiment production formalized in *MicroGen *system**. BIO.: Biologist; S.O.: Spotting Operator ; H.O.: Hybridisation Operator; P.O.: Processing Operator.

Besides problems related to a correct information management, our system also answers in many ways to the issue of supporting collaborative work. In fact, it eases information flow among different actors, and provides a mean to maintain constant knowledge about the current state of each performed experiment. An appropriate status flag characterizes each experiment and allows the actors involved in an experiment to check its current state at any time. An ideal experimental workflow, which models the real whole process of microarray experiment production, has been developed and implemented in *MicroGen *system (Figure 2). Such workflow follows experimental steps and supports control of their completeness and compliance to experimental procedures. This choice prevents incongruence and errors in the information management process while maintaining good flexibility in experiment production pipeline.

Finally, use of server-side web technologies, which allow centralization of data archiving and processing operations and easy deployment of suitable graphic user interfaces (GUI), enables *MicroGen *to easily provide collaborative work support also among different actors located in a distance. This choice makes faster and easier both managing and maintaining the system, and deploying the developed functionalities and GUIs to all its remote clients, besides lowering system maintenance costs. In fact, the employed web technologies allow using *MicroGen *everywhere an Internet/intranet access is available, requiring only a common web browser without any additional plug-in. The system is also simple to run. It only requires to be installed on a server computer with an Internet Information Server as web server.

*MicroGen *system is freely available for academic and non-profit use at:.

## Conclusion

*MicroGen *facilitates workflow management of spotted microarray experiment production, provides an efficient way to gather complete experimental information, and supports collaborative work. In fact, thanks to its well-defined core database architecture, *MicroGen *facilitates collection and storage of all experimental information according to the MIAME standard. Ordered availability of such information allows subsequent efficient and effective analyses of experimental results.

In addition to orderly store all information produced, by easing the process of information sharing, *MicroGen *represents a valid support for collaborative work even among research centres geographically distant from each other.

*MicroGen *also facilitates experimental data comparison. In fact, it allows saving quantitative results also in a standard text format. This increases portability and compatibility of results. Identification of results from experiment with similar characteristic is also facilitated thanks to the complete experimental information orderly stored within the system.

*MicroGen *graphic user interface is very simple and intuitive, providing an easy method for a biologist or a biotechnology technician to read or collect information about performed microarray experiments. The whole procedure of collecting experimental information is driven by the system, and all the steps to follow are simple and immediate. Forms with multiple choices or dynamic links are presented within web pages in order to quickly access a great number of different data. User's information, present progress status of an experiment, its MIAME data, and experimental results currently uploaded are all viewable at any time to all actors involved in the experiment.

## Methods

Using a relational database developed in MS-Access, we implemented a core data repository specifically structured to collect the whole information about all the actors involved in the production of spotted microarray experiments. The tables of this central database are grouped in three sections: a section containing information about the experiment itself and the additional files (such as quantification results) associated with the experiment; a section with the data about each user who worked on the experiment; and a MIAME section, which contains the whole information, according to the MIAME standard, about the experiment production (Figure 1). *MicroGen *core engine was developed using Microsoft Active Server Page technology, with Javascript language for scripting, and connections to the core repository were implemented by using Microsoft ActiveX Data Object technology. Hyper Text Markup Language 4.01 was used for formatting the graphic user interface implemented as web pages.

## Authors' contributions

SB and DC developed the whole *MicroGen *program and its core database engine, and wrote this paper. MM was responsible for project conception and coordination, developed clone's additional databases, and contributed to write this paper. FP provided supervision and funding of the project.

## Supplementary Material

Additional File 1 **MIAME compliant description of a microarray experiment. **PDF example file of the web page that *MicroGen *automatically generates with the whole MIAME compliant information collected about a specific spotted microarray experiment. Click here for file

## Figures and Tables

**Table 1:** *MicroGen *core database tables containing information of microarray experiment production according to the MIAME standard.

Experimental Design	• Experiment type • Experimental factors • Hybridisation design • Number of performed hybridisations • Type of used hybridisation references • Quality control steps • URL of websites with additional information related to the experiment
Biological Samples, Preparation Extraction and Labelling	• Origin • Characteristics • Manipulation • Extraction protocols • Labelling protocols • External controls
Array Design	• Platform type • Surface and coating specifications • PCR amplification • Array commercial availability • Spotting protocols • Additional treatments
Hybridisation Procedures and Parameters	• Hybridisation, blocking and washing protocols • Hybridisation, blocking and washing conditions
Measurement Data and Specification	• Scanning hardware and software • Image analysis software • Type of image quantifications • Measurement description
