# Supplementary material for: MicroGen: a MIAME compliant web system for microarray experiment information and workflow management
Source: BMC Bioinformatics. 2005 Dec 1;6(Suppl 4):S6. doi: 10.1186/1471-2105-6-S4-S6 (PMC1866379; doi:10.1186/1471-2105-6-S4-S6)
Supplement: Additional File 1 — MIAME compliant description of a microarray experiment. PDF example file of the web page that MicroGen automatically generates with the whole MIAME compliant information collected about a specific spotted microarray experiment. [file 1471-2105-6-S4-S6-S1.pdf]

Minimum Information About Microarray Experiments

Title of the selected experiment : ***"Transcriptional profiling studies provide direct evidence of diffuse coronary artery inflammation in diabetics that is attenuated by statins "***.

General Information

Date of registration : Wed Jan 12 11:27:20 UTC+0100 2005.

Type of density : Low Density.

Information stored in the labeling excel file.

Experimental Design

Type of experiment : our experiments examine gene expression profiles from normal and diseased human coronary arteries to gain new insights about the molecular and genetic basis of atherosclerosis.

Experimental factors : we compared differences in gene expression profiles between normal and diseased human coronary arteries. We also examined differences in gene expression in the presence or absence of risk factors for atherosclerosis, such as diabetes and hypertension. Finally, we compared differences in gene expression for samples obtained from patients treated versus those not treated with specific classes of cardiovascular medications, including aspirin, statins, ACE-inhibitors, and beta-blockers (Refer to Table 1 in the paper for further information).

The number of hybridizations performed in the experiment : a total of 103 hybridizations were performed on human coronary artery samples obtained from 17 patients.

The number of array needed in the experiment : 2.

The type of references used for the hybridizations : a 1:1 mixture of human umbilical vein endothelial cell (HUVEC) RNA and HeLa cell RNA was used as the reference RNA for all experiments. This combination of RNA was used after preliminary experiments demonstrated these cell types generated the greatest number of features with signal intensities that were statistically significant.

Hybridization design : all human coronary artery sample RNAs were hybridized against a common reference RNA sample.

Quality control steps taken : several dye swap experiments were performed for quality control purposes. However, due to the limited amount of total RNA obtained from each coronary artery segment, it was not possible to perform routine dye swaps or replicate experiments.

URL of any supplemental websites or database accession numbers : the website http://quertermous.stanford.edu contains additional information, including accession numbers, for all cDNA clones printed on our microarray.

Biological Samples

The origin of the biological sample : human coronary arteries were obtained from patients at Stanford University Hospital undergoing orthotopic heart transplantation.

Characteristics of the biological sample : basic demographic information, clinical risk factors, and medication profiles were obtained from each patient. Refer to Table 1 in the paper for a detailed description of patient characteristics.

Manipulation of biological samples : major epicardial coronary arteries were dissected from explanted hearts. The vessels were dissected longitudinally to expose the endoluminal surface and lesions were identified and scored by inspection using a dissecting microscope. The arteries were then divided into 1.0-2.0 cm normal and diseased segments.

Protocol for preparing the hybridization extract : samples were homogenized (PRO250 Homogenizer, 10 mm X 105 mm generator, PRO Scientific Inc., Oxford, Connecticut) in TRIzol (Invitrogen Life Technologies, Carlsbad, California). After phenol/chloroform extraction, the aqueous phase was applied to an RNeasy Mini column (Qiagen, Valencia, California) following manufacturer's instructions. Purity and quantity of total RNA was assessed using the RNA 6000 Nano Chip and Bioanalyzer (Agilent Technologies, Palo Alto, California).

Labeling protocol : 10 µg of total RNA was primed with 2 µl of 100µM $T_{16}N_2$ oligo d(T) DNA primer in a 50 ul reaction that was incubated at 70°C for 10 minutes. The RNA was reverse transcribed with Superscript II RT (Invitrogen Life Technologies, Carlsbad, California) to yield either cy3- or cy5-labelled double stranded cDNA. 50 µl of labelling mix and 50 µl of the oligo d(T) primed RNA were combined on ice and incubated at 42°C for 60 minutes. Final concentrations of labelling reagents were as follows: 10 mM DTT, 100 µM dNTP (without dCTP), 25 µM dCTP, 25 µM of cy3- or cy5-dCTP (NEN Life Science, Boston, Massachusetts), 1 µl RNase inhibitor per reaction, and 4U/µl SSII in 1x RT buffer. After labelling reaction, 2 µl of RNase A (0.05 mg/mL) was added to each 100 µl reaction and incubated for 30 minutes at room temperature to degrade residual RNA. Labelled cDNA targets were purified with a Qiaquick column (Qiagen, Valencia, California) following manufacturer's instructions. Purified targets were dried in a Speed Vac at 50°C until dry.

External controls (spikes) : Cy3/Cy5 HCV deposition control target (Operon, Qiagen, Valencia, California).

Array Design

Platform type : our cDNA microarrays were printed onto glass slides by Agilent Technologies using their proprietary SurePrint inkjet technology (Agilent Technologies, Inc., Palo Alto, CA).

Surface and coating specifications : surface and coating specifications were not provided by the manufacturer. Technical information is provided at the following website: http://www.chem.agilent.com/Scripts/PCol.asp?lPage=494 .

PCR amplification : the cDNAs spotted onto our arrays were chosen from cDNA libraries generated in our laboratory (see materials and methods section for details). Some cDNA clones were obtained commercially (Research Genetics, Carlsbad, California). All cDNA clones were amplified by PCR with primers specific for vector flanking sequences. PCR-amplified DNA was purified with automated column methodology (Qiagen, Valencia, California) and assessed by visualization on agarose gels.

Commercial availability of the array : Agilent Technologies SurePrint inkjet technology (Agilent Technologies, Inc., Palo Alto, CA). Technical information is provided at the following website: http://www.chem.agilent.com/Scripts/PCol.asp?lPage=494 .

Spotting protocol : spotting protocols were not provided by the manufacturer (Agilent Technologies, Inc., Palo Alto, CA).

Additional treatment : additional post-printing processing was not provided by the manufacturer (Agilent Technologies, Inc., Palo Alto, CA).

Hybridization Procedures and Parameters

Hybridization, blocking and washing conditions : cy3 or cy5 labelled targets were resuspended in 25 µl of hybridization cocktail and incubated in a water bath at 98°C for 2 minutes. Samples were then cooled for 15-20 minutes at room temperature. Hybridization cocktail contains the following reagents: 3 µl of a 1:1:1 mix of poly dA40-60 (8 mg/Ml) (Pharmacia, Peapack, New Jersey), human COT1 DNA (10 mg/ml) (Invitrogen, Carlsbad, California), and yeast tRNA (Invitrogen, Carlsbad, California); 12.5 µl of 2x deposition hybridization buffer (Agilent Technologies, Palo Alto, California); 2.5 µl of 10x cy3/cy5 HCV deposition control target (Operon, Qiagen, Valencia, California); and 7 µl water.

Hybridization, blocking and washing protocols : samples were quick spun and applied to our custom cDNA microarray (Agilent Technologies, Palo Alto, California), covered with a cover slip (Corning, Fountain Valley, California), placed in hybridization chambers (DieTech, San Jose, California) and incubated overnight (16-18 hours) at 65 °C. The arrays were washed with gentle stirring in 0.5X SSC/0.01% SDS for 5 minutes, then in 0.06X SSC for 10 minutes, 5 minutes, and 10 minutes (changing buffer after each wash). Arrays were spun dry at 1350 rpm at room temperature for 2 minutes.

Measurement Data and Specifications

Type of scanning hardware and software used : microarrays were scanned on an Agilent G2565AA Microarray Scanner System and raw images were quantified using Agilent Feature Extraction Software (Version A.6.1.1).

The quantitations based on the images : for further details about feature extracted data files, please use following link to Agilent's Feature Extraction Software PDF file: http://www.chem.agilent.com/scripts/cag_filexfer.asp?iWHID=CAG-05-099-00028703 .

The set of quantitations upon which conclusions are based : local background subtraction was performed and a LOWESS algorithm was used for data normalization. Dye normalization was performed to generate log ratios of sample signal/reference signal. This processed log ratio was then used for subsequent analysis with various statistical algorithms.

Type of image analysis software used : significance Analysis of Microarrays (SAM) software was used for data analysis (http://www-stat.stanford.edu/~tibs/SAM/) [Tusher, 2001 #2].

Description of measurements used in the analysis : the algorithm employs separate experiments to develop a measure of variance that is used to test whether observed differences in gene expression, in two sample type partitions, are likely to be real.

Notes on measurements (data selection and transformation procedures) : microarray data was also analyzed with the Threshold Number of Misclassifications (TNoM), a non-parametric score representing how well a gene separates two sample classes [Ben-Dor, 2001 #55; Ho, 2003 #115]. TNoM counts the minimal number of errors committed by using a threshold on the expression values for this separation.

Download Section

Available images of the arrays and available excel files of the processed images:

**Array n° 1**	Date of its creation: Wed Jan 12 11:31:20 UTC+0100 2005
	Image from the CY3 channel<br>Excel file of its left part<br>Excel file of its left part
	Image from the CY5 channel<br>Excel file of its left part<br>Excel file of its left part

**Array n° 2**	Date of its creation: Wed Jan 12 11:31:20 UTC+0100 2005
	Image from the CY3 channel<br>Excel file of its left part<br>Excel file of its left part
	Image from the CY5 channel<br>Excel file of its left part<br>Excel file of its left part

People involved in the experiment:

Biologist

Spotting Operator

Hybridization Operator

Processing Operator
